# Computed tomography-guided percutaneous drainage of tension pneumomediastinum

**DOI:** 10.1590/0100-3984.2021.0065

**Published:** 2022

**Authors:** Paula Nicole Vieira Pinto Barbosa, Flávio Scavone Stefanini, Almir Galvão Vieira Bitencourt, Jefferson Luiz Gross, Rubens Chojniak

**Affiliations:** A.C.Camargo Cancer Center, São Paulo, SP, Brazil.

## INTRODUCTION

Pneumomediastinum was frst described in 1819 by René Laennec as a secondary complication of thoracic trauma. The clinical presentation ranges from mild symptoms, such as cough, dyspnea, and chest pain, to severe symptoms, including acute respiratory impairment with hypoxemia, as well as decreased venous return in cases of tension mediastinal emphysema(^1^,^2^).

In hospitalized patients, the main cause of pneumomediastinum is mechanical ventilation with high peak airway pressure and positive end-expiratory pressure. The combination of high pressure indices in the mechanical ventilation parameters and acute infammation of the lung parenchyma, whether by viral, bacterial or even drug-related lung disease, is a major risk factor for air leakage into the fat planes of the mediastinum, with a restrictive ventilatory defect, which can progress to a potentially fatal condition. Although tension pneumomediastinum is a rare diagnosis, it has been observed in critically ill patients with coronavirus disease 2019(^3^, ^4^, ^5^).

Computed tomography (CT) of the chest is the best method for identifying pneumomediastinum and its complications. Recognizing pneumomediastinum on a chest X-ray can be challenging, especially when there is a limited volume of air in the mediastinum or when extensive subcutaneous emphysema is superimposed on thoracic structures on X-rays taken at the bedside.

## PROCEDURE

In patients with pneumomediastinum, treatment should be started immediately, before there is clinical evidence of cardiac tamponade or increased intracranial pressure. Invasive surgical techniques classically described for the treatment of pneumomediastinum include tracheostomy, sternotomy, and mediastinotomy, all of which carry high risks of complications (hemothorax, infection, or inadvertent injury to neighboring structures) for patients with severe cardiopulmonary impairment. Recent studies have described minimally invasive techniques that are faster, are more efficacious, and have lower operative risks(^6^, ^7^, ^8^), such as intermittent needle aspiration at the bedside and percutaneous mediastinal tube drainage under CT or fuoroscopy guidance.

**Figure 1:**
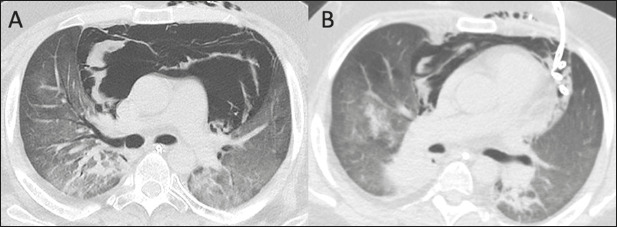
A 22-year-old male patient diagnosed with atypical extraventricular neurocytoma, undergoing treatment for pneumocystosis, who evolved to severe hypoxemia complicated by possible bronchial aspiration. **A:** Chest CT scan showing extensive pneumomediastinum with a tension aspect, dissecting the mediastinal fat planes and exerting a compressive effect on both lungs. **B:** Percutaneous CT-guided drainage was performed as an emergency procedure, under general anesthesia, with insertion of a 14F Wayne drainage catheter through the left anterior chest wall, with immediate reduction of the pneumomediastinum and lung reexpansion.

**Figure 2:**
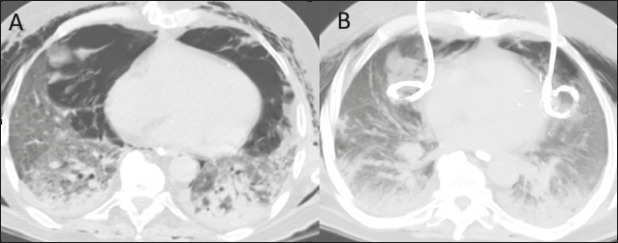
A 74-year-old male patient with a history of metastatic renal carcinoma who presented with coronavirus disease 2019 and required endotracheal intubation with mechanical ventilation. **A:** Chest CT scan acquired two days after endotracheal intubation, showing subcutaneous emphysema and tension pneumomediastinum, with no visible damage to the tracheal wall. **B:** CT-guided bilateral percutaneous drainage, performed by placing two 14F Wayne drainage catheters in the largest air loculi, on each side of the anterior and medial mediastinal compartments, which resulted in immediate reduction of the pneumomediastinum, allowing greater expansion of the lung parenchyma.

In the cases illustrated here, CT-guided percutaneous mediastinal drainage was performed under sedation by inserting a 14F percutaneous pneumothorax drainage catheter (Wayne Pneumothorax Catheter; Cook Medical, Bloomington, IN, USA) into the largest loculated air collection in the mediastinum (Figure 1). The CT guidance makes it possible to avoid damage to blood vessels, the lung parenchyma, and other important structures along the path of the catheter. After the drain had been placed, it was connected to a one-way (Heimlich) valve to direct the air out of the chest. Post-procedural (follow-up) CT scans showed an immediate reduction in the pneumomediastinum, allowing greater expansion of the lung parenchyma. If necessary, more than one drain can be inserted for more effective drainage (Figure 2). Although infrequent, potential complications of the procedure include bleeding and pneumothorax.

## CONCLUSION

In patients with acute infammatory involvement of the lung parenchyma, endotracheal intubation with high ventilatory pressure parameters can trigger tension pneumomediastinum with severe cardiopulmonary involvement. Minimally invasive percutaneous interventional treatment with insertion of mediastinal drains is safe and effective for emergency decompression, providing immediate improvement in ventilatory and hemodynamic parameters.

## References

[1] Dionísio P, Martins L, Moreira S (2017). Spontaneous pneumomediastinum: experience in 18 patients during
the last 12 years. J Bras Pneumol.

[2] Dajer-Fadel WL, Argüero-Sánchez R, Ibarra-Pérez C (2014). Systematic review of spontaneous pneumomediastinum: a survey of
22 years’ data. Asian Cardiovasc Thorac Ann.

[3] Brogna B, Bignardi E, Salvatore P (2020). Unusual presentations of COVID-19 pneumonia on CT scans with
spontaneous pneumomediastinum and loculated pneumothorax: a report of two
cases and a review of the literature. Heart Lung.

[4] Campisi A, Poletti V, Ciarrocchi AP (2020). Tension pneumomediastinum in patients with
COVID-19. Thorax.

[5] Wali A, Rizzo V, Bille A (2020). Pneumomediastinum following intubation in COVID-19 patients: a
case series. Anaesthesia.

[6] Dondelinger RF, Coulon M, Kurdziel JC (1992). Tension mediastinal emphysema: emergency percutaneous drainage
with CT guidance. Eur J Radiol.

[7] Argirò R, Di Donna C, Morosetti D (2021). CT-guided emergency drainage of tension pneumomediastinum in a
young patient with acute lymphoid leukemia and aspergillus fumigatus
pulmonary infection. J Bronchology Interv Pulmonol.

[8] Chon KS, vanSonnenberg E, D’Agostino HB (1999). CT-guided catheter drainage of loculated thoracic air collections
in mechanically ventilated patients with acute respiratory distress
syndrome. AJR Am J Roentgenol.

